# Role of microbial and chemical composition in toxicological properties of indoor and outdoor air particulate matter

**DOI:** 10.1186/s12989-014-0060-6

**Published:** 2014-11-25

**Authors:** Mikko S Happo, Olli Sippula, Pasi I Jalava, Helena Rintala, Ari Leskinen, Mika Komppula, Kari Kuuspalo, Santtu Mikkonen, Kari Lehtinen, Jorma Jokiniemi, Maija-Riitta Hirvonen

**Affiliations:** Department of Environmental Science, University of Eastern Finland, P.O. Box 1627, FI-70211 Kuopio, Finland; Mikrobioni Oy, Microkatu 1, P.O. Box 1188, FI-70211 Kuopio, Finland; Finnish Meteorological Institute, Atmospheric Research Centre of Eastern Finland, P.O. Box 1627, FI-70211 Kuopio, Finland; Department of Applied Physics, Kuopio, University of Eastern Finland, P.O.Box 1627, FI-70211 Kuopio, Finland; VTT Technical Research Centre of Finland, Fine Particles, Espoo, P.O. Box 1000, FI-02044 Espoo, Finland; Department of Environmental Health, National Institute for Health and Welfare, P.O. Box 95, FI-70701 Kuopio, Finland

**Keywords:** Particulate matter, Inflammation, Cytotoxicity, Microbes, Indoor air PM, Ambient air PM, Chemical composition

## Abstract

**Background:**

Ambient air particulate matter (PM) is increasingly considered to be a causal factor evoking severe adverse health effects. People spend the majority of their time indoors, which should be taken into account especially in future risk assessments, when the role of outdoor air particles transported into indoor air is considered. Therefore, there is an urgent need for characterization of possible sources seasonally for harmful health outcomes both indoors and outdoors.

**Methods:**

In this study, we collected size-segregated (PM_10–2.5_, PM_2.5–0.2_) particulate samples with a high volume cascade impactor (HVCI) simultaneously both indoors and outdoors of a new single family detached house at four different seasons. The chemical composition of the samples was analyzed as was the presence of microbes. Mouse macrophages were exposed to PM samples for 24 hours. Thereafter, the levels of the proinflammatory cytokines, NO-production, cytotoxicity and changes in the cell cycle were investigated. The putative sources of the most toxic groups of constituents were resolved by using the principal component analysis (PCA) and pairwise dependencies of the variables were detected with Spearman correlation.

**Results:**

Source-related toxicological responses clearly varied according to season. The role of outdoor sources in indoor air quality was significant only in the warm seasons and the significance of outdoor microbes was also larger in the indoor air. During wintertime, the role of indoor sources of the particles was more significant, as was also the case for microbes. With respect to the outdoor sources, soil-derived particles during a road dust episode and local wood combustion in wintertime were the most important factors inducing toxicological responses.

**Conclusions:**

Even though there were clear seasonal differences in the abilities of indoor and outdoor air to induce inflammatory and cytotoxic responses, there were relatively small differences in the chemical composition of the particles responsible of those effects. Outdoor sources have only a limited effect on indoor air quality in a newly built house with a modern ventilation system at least in a low air pollution environment. The most important sources for adverse health related toxicological effects were related to soil-derived constituents, local combustion emissions and microbes.

**Electronic supplementary material:**

The online version of this article (doi:10.1186/s12989-014-0060-6) contains supplementary material, which is available to authorized users.

## Background

Exposure to particulate matter (PM) was recently estimated by the European Topic Centre on Air and Climate Change (ETC/ACC) to be responsible for more than 455,000 premature deaths annually in the EU countries. Moreover, anthropogenic outdoor air fine PM (PM_2.5_; <2.5 μm) has been estimated to cause on average of 2.1 million premature deaths worldwide every year [1]. Airborne particles are also known to pose a major health risk to susceptible population groups such as patients with chronic respiratory or cardiovascular disease, asthmatics, the elderly and children [2,3]. On the basis of epidemiological and toxicological studies, it is clear that the chemical composition of PM has an essential role when considering their health impacts [4,5]. The chemical composition of the particles is also affected by several factors, such as season, meteorology, geographical location, photochemical transformation and vicinity of emission source. It has been shown in toxicological studies that the seasonal variation in the PM composition has a major effect on its toxicological properties [6-11]. However, there still remain large uncertainties about the role of exposure to indoor versus outdoor air particles, especially in regard to their chemical characteristics and their sources. In most of the study setups, the role of outdoor air PM has been characterized thoroughly, whereas the role of exposure to indoor air particles has been poorly explored, even though people who are exposed to particles spend the majority (>80%) of their time indoors in work, at home, in or near a vehicle [12] and only a mere 5.6% of their time outdoors [13].

It is a challenge to separate the relative contributions of indoor and outdoor sources of particles especially when an individual’s exposure is being estimated. Outdoor air is a complex mixture of different sized solid and liquid particles originating from a huge number of anthropogenic and natural sources. On top of that, exposure includes indoor air pollutants originating from primary and secondary sources such as combustion, cooking, tobacco smoke, cleaning and usage of water e.g. for during showering [14,15], and also from outdoor-to-indoor transported pollutants. The outdoor-to-indoor infiltration rate is largely dependent on the ventilation system and ventilation conditions [16]. Moreover, there are also size related differences in the abilities of particles to gain access to the indoor air. The largest thoracic coarse particles (PM_10–2.5_; 2.5 μm < D_p_ <10 μm) are filtrated or they rapidly deposit on surfaces during the house air intake [14,17]. Fine particles (PM_2.5_, D_p_ <2.5 μm) have a high infiltration rate, but in contrast, the smallest ultrafine particles (PM_0.1_, D_p_ <0.1 μm) have low infiltration rates due to diffusion driven deposition during air intake [16]. Fine particles contain combustion-derived carbon (soot), metals and salts, secondary inorganic components and organic matter [14,18]. In a study conducted in residents in several U.S. cities 25% of indoor PM_2.5_ could not be linked with any known sources [13]. Moreover, those sources of indoor generated particles are dependent on different factors e.g. air exchange rate, building materials, building age, floor material and family and domestic characteristics [19].

Microbes and non-infectious bioaerosols have been the best studied contaminants of indoor air and they can evoke a large variety of adverse health effects such as respiratory symptoms, frequent respiratory infections, asthma and clusters of autoimmune diseases [15,20]. However, the role of microbes in the outdoor air particles in evoking adverse health effects has not been extensively studied. Most of the studies have focused on the role of endotoxins in inducing inflammatory activity. Microbes and their metabolites are known not only to have different toxic potencies and capabilities of inducing the production of inflammatory mediators [21-24], but they also undergo interactions leading to upregulation and downregulation of the induced responses [25,26]. The role of chemical constituents of the indoor air particles in induced toxicological responses is not clear, largely because of methodological limitations [27]. For example insufficient particle masses may not permit both chemical and toxicological analyses, because low-volume impactors are often used for sample collection. Another widely used method is the collection of settled dust, but these samples cannot be classified by size and they also become easily contaminated.

In attempt to overcome these challenges, we have applied a high-volume sampling technique in the sample collections both indoors and outdoors simultaneously, which guaranteed that we could obtain a sufficient amount of particle mass within a reasonable sampling duration to allow performance of a wide variety of chemical, microbiological and toxicological analyses on each sample. In this study set-up, the samples were collected in parallel from the indoor and outdoor air of a new single family house in four different seasons. The samples were characterized for their chemical and microbial properties [28] and their effects on inflammation, cytotoxicity as well as changes in the cell cycle were examined [29]. It was found that the PM_10_ mass concentrations indoors and outdoors were nearly at the same level, but they differed in their chemical and microbial contents in terms of particle size-ranges, seasons as well as between indoor and outdoor air. PM_10–2.5_ particles of the warm seasons (spring, summer) had the highest amount of metals that are typical for soil-derived dust, but also the highest microbe concentrations. The indoor air concentration of microbes did not reveal such clear variations. Instead, PM_2.5–0.2_ particles gathered during warm seasons had the highest metal concentrations typically released during biomass burning (K, Zn, Pb, Fe) and had originated from long range transported particles of wildfires. However, the PAH-concentrations were the highest during wintertime, which indicates that local residential heating was a major source of PAHs in the studied site. In the summertime, PAH concentrations were the lowest, most probably due to the rapid photodegradation of the compounds. The toxicological analyses revealed that outdoor air particles were inducing higher inflammatory responses than the corresponding indoor air samples, whereas indoor air particles were associated with a higher potency to induce cytotoxicity in mouse macrophages. Particles in both coarse and fine size-ranges collected during warm seasons had a higher inflammatory potency than those collected during the wintertime sampling campaign.

In an attempt to reveal chemical constituents and microbes that were responsible for the detected toxicological responses, we subjected this combined large multidisciplinary data set to a statistical analysis which included in-depth data from four seasonal sampling campaigns focusing on chemical and microbiological PM characteristics [28] and toxicological properties [29] of the very same PM samples. The aim of this study was to reveal possible connections between the chemical constituents and microbe concentrations and induced toxicological responses by using principal component analysis (PCA) and Spearman’s correlation test. Principal component analysis (PCA) was used to determine the most toxic groups of components, whereas Spearman correlation analysis was used to detect the role of single constituent or microbe. In summary, these results described one way to identify the most plausible particle sources that are behind the toxicological responses. This information is valuable when evaluating the indoor air quality of modern buildings and the most harmful sources of its impurities.

## Results and discussion

This study revealed several clear associations between different particulate sources in different seasons and induced toxicological responses. Here in this work, we provide new information about role of outdoor sources in the indoor air quality from a toxicological point of view. This is the first work which has included an extensive chemical characterization of the particulate samples both indoor and outdoor, including microbes and compared them to the evoked toxicological responses. It also provides new insights into the roles of coarse and fine particulate size ranges as well as on the role of seasonal variation in the toxicological potencies of the gathered particles. This is of special importance since it was found that the outdoor air quality has only a very limited influence on the indoor air quality in a modern house leaving unanswered several questions concerning the characterization of the sources behind these responses [29].

Despite the relatively large sampling volumes, the collected particulate mass in some of the size fractions was still too small to carry out all desired chemical and toxicological analyses, which was a problem in some of the statistical analyses. Therefore, analyses were made by combining or excluding different variables, such as particle size, season or indoor/outdoor origin of particulate samples. The correlation analyses were made using data from exposures to PM_10–2.5_ and PM_2.5–0.2_ samples, because the PM_0.2_ samples induced mostly negligible and statistically insignificant responses. Spearman correlation coefficients between detected responses and chemical components behaved similarly with all of particle dose levels used in this study. Therefore, all of the responses from different dose levels were used in the analysis in an attempt to have a high enough number of samples to satisfy the statistical analyses.

The detailed description of the chemical composition of the samples has been presented in Sippula et al. [28] and their toxicological responses reported by Happo et al. [29] and collected to Additional files 1 and 2. Briefly, despite the outdoor-to-indoor-transport of the particles, it was found that indoor sources dominated the toxicological responses of the particles found inside the house. Indoor air particles exhibited a greater ability to reduce cell viability than the corresponding outdoor air samples. With respect to outdoor samples, those collected during warm seasons had the highest inflammatory activity. Snow coverage, as well as the limited photochemical transformation in wintertime had a clear effect on the characteristics of outdoor air particles. In the spring, a road dust episode greatly increased the proportion of soil-derived mineral particles. In PM_2.5–0.2_, the highest metal concentrations were observed in the summertime, when the air quality was affected by long distance transported smoke from two episodes of major Russian wildfires which were burning at a distance approximately 1000 km away so that the smoke appeared after 1–2 days of air mass travel time [30]. However, PAH concentrations were at their highest during wintertime, mostly because of local residential heating. In the summertime, their concentrations were not increased, mostly because of the extensive photochemical transformation. Microbial DNA concentrations were highest in the coarse size fraction, and their peak levels occurred in wintertime indoors and in summertime outdoors.

### Principal component analysis

Urban air and indoor air pollutants are a complex mixture of solid and liquid particles originating from distinct sources. Therefore, it is almost impossible to state which particular component would be responsible for the adverse health effects. However, it is possible to estimate their contributions by using statistical methods such as principal component analysis (PCA) to identify groups of chemical components in PM that have similar correlations with the observed responses. During the first steps of our PCA, different subgroups (indoor and outdoor, PM_10–2.5_ and PM_2.5–0.2_) were tested separately. When taking into account the uncertainty caused by the small number of samples in subgroups, the results were similar to those obtained with the combined data and thus we chose to use the combined data in the formation of the PC’s. The highest statistical significances were observed when the toxicological responses to the dose of 150 μg/ml were used in the PCA. On the basis of those calculations, we were able to establish four groups that display high similarities to selected particulate induced inflammatory or cytotoxic responses. The resulting PC’s were used as predictors in a linear regression analysis intended to define their impact on the toxicological responses. The results of this regression analysis are shown in Table 1.

**Table 1:** **The results of the regression analysis where the toxicological responses were predicted with the principal components (PC)**

**Indoor air**	**Predictors**	**B-value**	**Std. Error**	**P-value**	**R Square**
MTT	PC1	23.200	7.540	.012	.486
PI	PC1	25.545	7.361		
	PC3	−16.478	5.672	.027	.663
	PC4	.002	.001		
SubG_1_	PC2	3.790	1.965	.083	.271
TNF-α	PC1	2361.993	528.044	.002	.754
	PC2	−1030.431	320.505		
MIP-2	PC1	13903.703	4714.166	.007	.672
	PC2	−6730.010	2861.338		
**Outdoor air**					
MTT	PC2	4.983	3.545	.010	.733
	PC4	.002	.001		
PI	PC2	1.718	1.538	.013	.708
	PC4	.001	.000		
SubG_1_	PC1	−4.451	1.108	.037	.829
	PC2	−1.273	.748		
	PC3	7.641	2.024		
	PC4	.000	.000		
TNF-α	PC1	129.943	323.975	.000	.955
	PC4	.642	.063		
MIP-2	PC1	8471.839	3498.369	.000	.577
	PC4	1.783	.601		

Columns in the table show independent predictors for each dependent, regression coefficient (B) and its standard error, significance of the total model (p-value) and the coefficient of determination (R^2^), respectively. Indoor (*n* = 18) and outdoor air (*n* = 15) were tested separately.

The first group (hereafter PC1) contained Al, Ca, Fe, K, Mg, Na and Ni, which are elements mostly originating from soil and to some extent from non-exhaust particulate matter attributable to road traffic [31]. This view is supported by the data showing clearly their highest concentrations in the PM_10–2.5_ outdoor samples in the springtime during the road dust episode [28]. In the PCA, it was found that soil related constituents in the indoor samples increased disturbances in mitochondrial activity and cytotoxicity index, while no such association was found in the samples collected outdoors. It is possible that the source of indoor air cytotoxicity was some unknown source other than the soil-derived components. This is supported by the study of [32] with rat alveolar macrophages, where no differences were found in cytotoxicity between the PM_2.5_ samples collected either during a dust storm or in a normal day in Baotou City, China. PM with a high organic content has induced apoptotic effects [33]. Instead, the soil-derived particles from the outdoors gathered in this study were linked to a reduced number of apoptotic cells, which may be explained by the previous findings that the cytotoxicity is dominated by the short term effects such as mitochondrial toxicity and necrosis when macrophages are exposed to particles with a high mineral content e.g. [8]. In agreement with several previous studies [33-35], soil-derived components in both the indoor and outdoor PM were associated with clear increase in the inflammatory responses.

The second group of chemical components (PC2) that displayed some similarities in the PCA was SO_4_, NO_3_, V, Zn and the total sum of PAH compounds. Those constituents are also closely linked to the outdoor source, because their highest concentrations were detected in the PM_10–2.5_ and PM_2.5–0.2_ outdoor samples from wintertime. Most probably these originate from a mixture of long range transported aged particles (SO_4_, NO_3_) and local residential heating with wood combustion (Zn, total PAHs) and oil combustion [36]. Their toxicological activities are known to vary largely depending on their concentration as well as on seasonal changes. PC2 components exhibited increasing cytotoxicity in both MTT-analysis as well as in the PI exclusion assay with outdoor air samples, where their concentrations were mostly higher than in the corresponding indoor air samples. However, the role of PC2 components in apoptotic cells was bidirectional i.e. the indoor air had a positive B-value whereas the outdoor air samples showed negative B-values, which may indicate that there is some other existing mutual factor. In the indoor air, this may also be linked to the decreased inflammatory activity induced by some of PC2 components. This has been seen in several previous studies where PAH-rich samples have been investigated [33,35,37]. PAH-compounds are known to induce immunosuppression and exert genotoxic properties, which may also be reflected in the increased number of apoptotic cells [38]. However, some water-soluble metal-rich PM extracts have also been postulated to induce more oxidative DNA damage than organic compounds [39], and furthermore poorly soluble particles may have role in inducing secondary genotoxicity [40].

The third group (PC3) included Cl, F and Cu. However, this group produced some inconsistent associations with the responses, which caused some uncertainties about their possible sources. The PC3 group components in the indoor air reduced disturbances in the functionality of the cell membrane. In outdoor air samples, PC3 components increased the number of apoptotic cells. It is possible that this factor have some connection to a specific source, which may in some way explain those associations.

The fourth group (PC4) that displayed some statistical significances in PCA included only total bacteria (Bact). Thus the effect of total bacteria will be discussed as an individual variable not as a principal component. In the indoor samples, total bacteria only exerted an effect visible in the PI exclusion assay, whereas in the outdoor air samples it was found to be capable of inducing cytotoxicity, apoptotic activity as well as inflammation. Bacteria, especially gram negative bacteria with endotoxins, are known to have strong toxicological effects in macrophages [41].

### Spearman correlation analyses

Those groups of chemical constituents that were found to result in statistically significant values in the PCA were investigated in detail by using Spearman correlation coefficients. There clear similarities in the responses of the two inflammatory markers, TNF-α and MIP-2, and therefore only TNF-α will be discussed as a marker of inflammation to prevent repetition. In addition, the MTT-test was also selected, because it was clearly a more sensitive marker of cytotoxicity than the PI-staining method, and revealed larger differences between the samples. Instead, in the cell cycle analyses, only SubG_1_, i.e. the marker of apoptotic cells was found to correlate statistically significantly with the chemical constituents, whereas G_2_/M phase of the cell cycle had only negligible associations with chemical composition of the samples, indicating that none of the samples significantly disrupted the normal cell cycle.

In the Spearman analyses, all the doses that were used in the exposures were included in the calculations to obtain as large a number of measurements (*n*) as possible. In addition, all the dose levels were tested separately, but their results were found to be nearly identical with the analysis conducted with all measurements (data not shown). The calculated coefficients for correlations of chemical constituent when all the measured conditions were combined with the measured toxicological responses in the mouse macrophages are shown in Table 2.

**Table 2:** **Associations between inorganic soluble chemical constituents, polyaromatic hydrocarbons (PAHs) and microbes in PM samples and the inflammatory and cytotoxic response markers in mouse macrophages after exposure to three different doses of particles (50, 150 and 300 μg/ml) for 24 h**

		**n**	**MTT**	**PI**	**SubG**_**1**_	**TNFα**	**MIP2**
PC1	Al	48	.584**	.487**	0.031	.786**	.808**
	Ca	48	.725**	.620**	.391**	.619**	.687**
	Fe	45	.595**	.496**	−0.013	.816**	.805**
	K	48	.676**	.660	.440**	.492**	.507**
	Mg	48	.686**	.639**	0.256	.680**	.720**
	Na	48	.681**	.605**	.324*	.568**	.608**
	Ni	48	.472**	.461**	.349*	.298*	.305*
PC2	SO_4_	48	0.059	0.138	.456**	.-468**	.-440**
	NO_3_	45	.383**	.362*	.341*	0.147	0.22
	V	48	.505**	.468**	.468**	0.194	0.239
	Zn	48	.486**	.478**	.557**	0.104	0.154
	TotalPAH	48	0.004	−0.026	.420**	.-499**	.-416**
PC3	CI	48	.686**	.503**	.381**	.554**	.616**
	F	39	.767**	.670	.744**	0.29	.345*
	Cu	48	.621**	.662**	.485**	.409**	.446**
PC4	Universal bacterial assay	48	.812**	.649**	.327*	.669**	.677**
Other	Genotoxic PAH	48	−0.027	−0.038	.411**	.-539**	.-466**
	EU PAH	48	−0.108	−0.073	.404**	.-626**	.-571**
	*Penicillium sp./Aspergillus sp.*	48	.702**	.620**	.320*	.610**	.616**
	*Cladosporidium cladosprioides*	45	.687**	.619**	0.201	.789**	.766**
	*Streptomyces sp.*	39	.573**	.604**	.615**	0.072	0.09
	*Mycobacterium sp.*	39	.807**	.762**	.499**	.748**	.768**

The values are Spearman correlation coefficients (ρ), (** *p* < 0.01, * *p* < 0.05).

Genotoxic PAH compounds according to WHO (1998).

EU PAH compounds according to the EU Directive 2004/107/EC.

**Figure 1:**
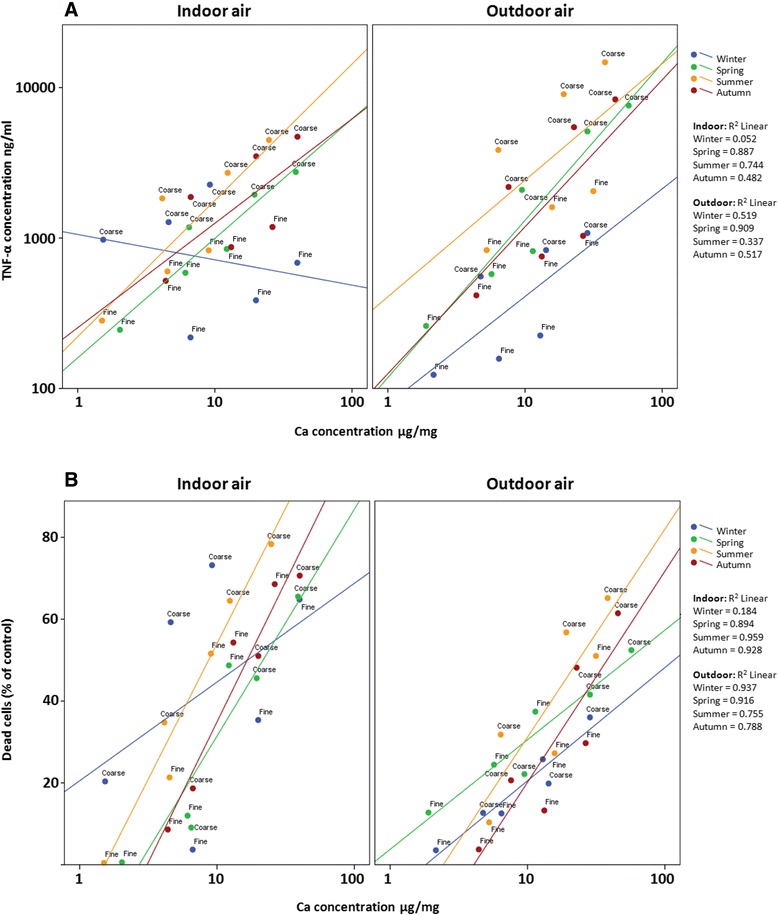
**The correlation between calcium (PC1, soil related constituents) concentration in the PM**_**10–2.5**_ **and PM**_**2.5–0.2**_ **samples and inflammatory (A; TNF-α) and cytotoxicity (B; MTT) responses in mouse macrophages after exposure to three different doses (50, 150 and 300 μg/ml for 24 h) of indoor or outdoor air PM samples.** The Pearson correlation coefficients are included in the figure panel. Seasons are differentiated by colors (blue = winter, green = spring, yellow = summer, red = autumn). X-axis and Y-axis **(A)** are both in logarithmic scales whereas Y-axis **(B)** in linear scale.

When all the samples were analyzed together, the PC1 group’s constituents were statistically significantly associated with increased cytotoxic activity and inflammation (Figure 1). However, their role in apoptosis was not so clear, because Al, Fe and Mg were not found to induce any clear effect on the SubG_1_ phase. On the basis of several previous studies, soil-derived constituents are known to be powerful inducers of inflammation and cytotoxicity [7,33,35]. However, their role in cytotoxicity is most likely to be mediated via necrosis and their role to evoke apoptosis was inconsistent.

**Figure 2:**
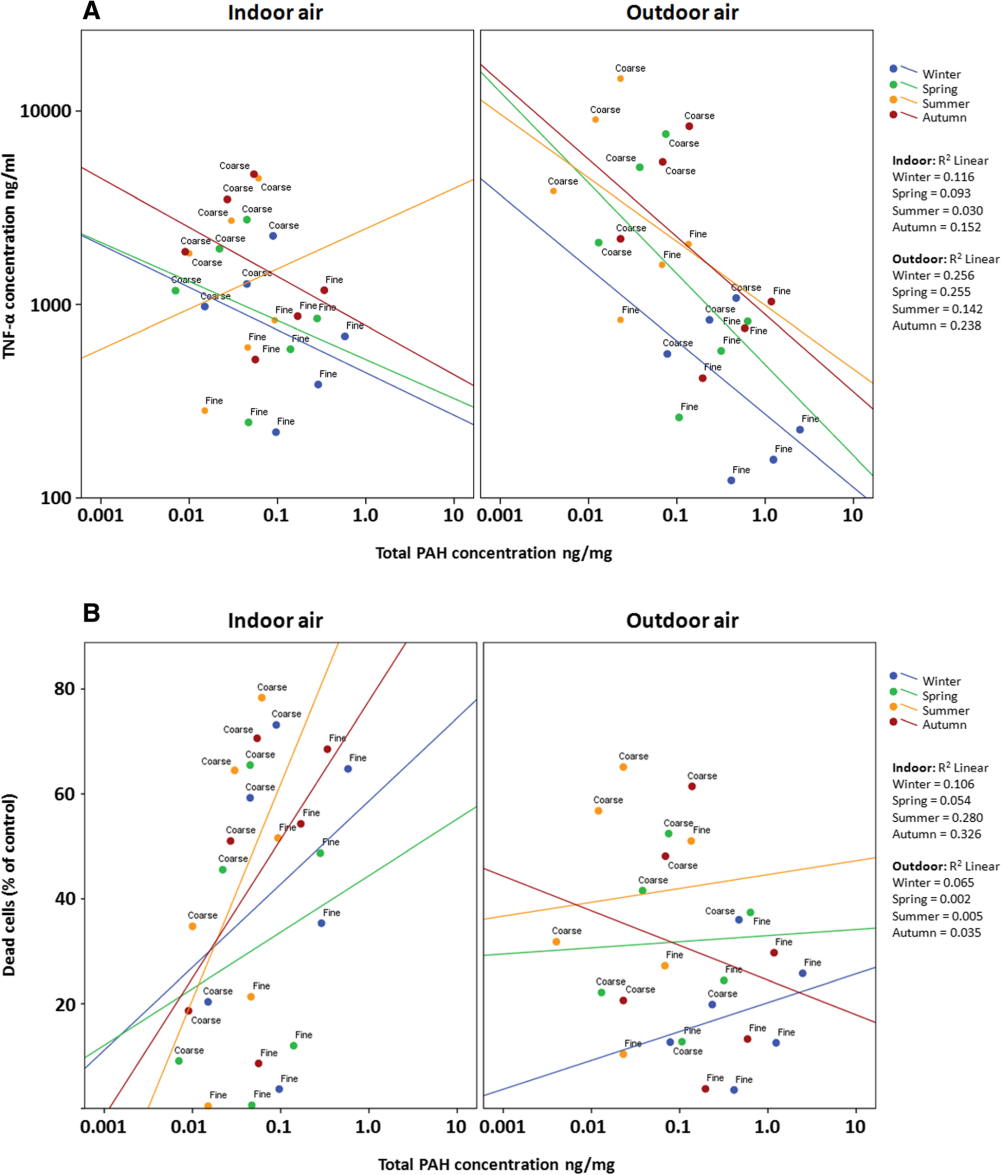
**The correlation between total PAH concentration (PC2, local combustion related constituents) in the PM**_**10–2.5**_ **and PM**_**2.5–0.2**_ **samples and inflammatory (A; TNF-α) and cytotoxicity (B; MTT) responses in mouse macrophages after exposure to three different doses (50, 150 and 300 μg/ml for 24 h) of indoor or outdoor air PM samples.** The Pearson correlation coefficients are shown in the figure panel. Seasons are differentiated by colors (blue = winter, green = spring, yellow = summer, red = autumn). X-axis and Y-axis **(A)** are presented in logarithmic scale and Y-axis **(B)** in linear scale.

In contrast, PAH-compounds, SO_4_ and NO_3_ exhibited a negative correlation with the inflammatory markers (Figure 2). Interestingly, PAHs and SO_4_ did not correlate with the cytotoxicity markers, but showed a positive association with the SubG_1_ phase, indicating that they were influencing the early apoptotic rather than late apoptotic or necrotic cell death. A comparison of this result to the PC1 correlations reveals a clear difference in the cell death mechanisms being activated by these different groups.

**Figure 3:**
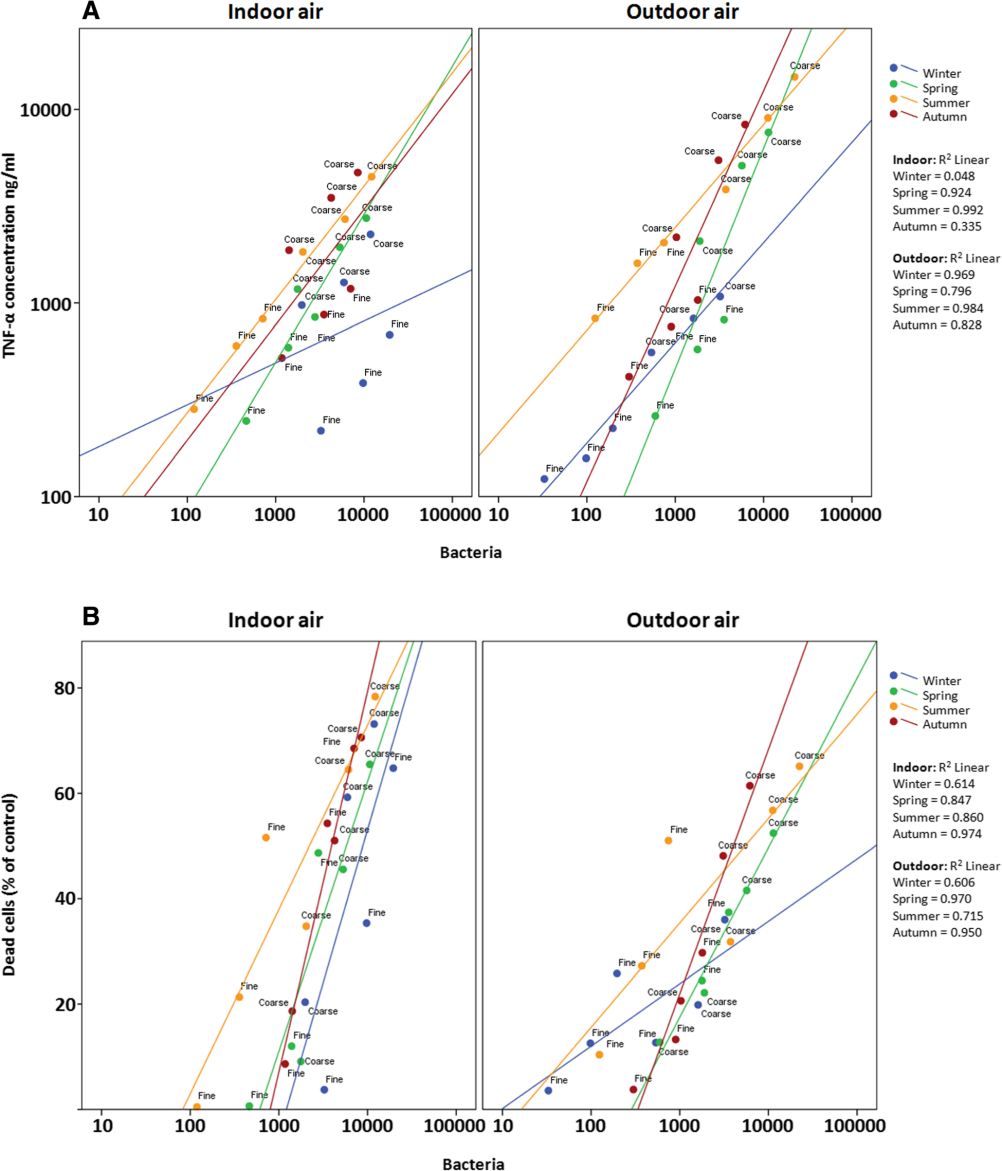
**The correlation between total bacteria concentration in the PM**_**10–2.5**_ **and PM**_**2.5–0.2**_ **samples and inflammatory (A; TNF-α) and cytotoxicity (B; MTT) responses in mouse macrophages after exposure to three different doses (50, 150 and 300 μg/ml for 24 h) of indoor or outdoor air PM samples.** The Pearson correlation coefficients are included in the figure panel. Seasons are differentiated by colors (blue = winter, green = spring, yellow = summer, red = autumn). X-axis and Y-axis **(A)** are presented in logarithmic scale and Y-axis **(B)** in linear scale.

Most of the detected microbes had a positive correlation with all of the detected responses (Figure 3), excluding *Streptomyces sp*. that did not correlate with the inflammatory responses and *Cladosporidium cladosprioides*, which showed no correlation with SubG_1_ phase in the cell cycle. Similarly, most of the soil related chemical constituents exhibited also positive correlations with the measured responses. Soil related constituents are known to be prevalent in the PM_10–2.5_ particles that also have higher endotoxin levels [42,43]. Therefore it is possible that microbial content and soil-derived particulate material share, at least in part, their abilities to exert toxicological responses.

#### Comparison of indoor and outdoor air samples

The calculated coefficients for the correlations of separately analyzed indoor and outdoor samples are shown in Table 3. The findings in this analysis supported the results obtained from PCA and were well in line with the results of our previous studies with size-segregated ambient air particles [33,35].

**Table 3:** **Associations between inorganic soluble chemical constituents, polyaromatic hydrocarbons (PAHs) and microbes in indoor and outdoor air PM separately and the inflammatory and cytotoxic response markers in mouse macrophages after exposure to three different doses of particles (50, 150 and 300 μg/ml) for 24 h**

**Indoor**													
		**n**	**MTT**	**PI**	**SubG**_**1**_	**TNFα**	**MIP2**	**n**	**MTT**	**PI**	**SubG**_**1**_	**TNFα**	**MIP2**
PC1	A1	24	.546**	.457*	0.302	.722**	.713**	24	.912**	.735**	−0.094	.892**	.921**
	Ca	24	.771**	.637**	.682**	.543**	.603**	24	.856**	.733**	0.209	.730**	.777**
	Fe	21	.640**	.548*	0.287	.884**	.830**	24	.901**	.719**	−0.104	.882**	.909**
	K	24	.809**	.777**	.737**	.488*	.499*	24	.785**	.726**	0.285	.590**	.597**
	Mg	24	.589**	.555**	.528**	.453*	.477*	24	.928**	.767**	−0.026	.887**	.915**
	Na	24	.661**	.646**	.599**	.484*	.514*	24	.888**	.645**	0.13	.651**	.695**
	Ni	24	.510*	.540**	.507*	0.306	0.307	24	.623**	.504*	0.369	0.307	0.311
PC2	SO_4_	24	0.273	.467*	.583**	−0.357	−0.298	24	−0.12	−0.164	0.403	.-543**	.-529**
	NO_3_	24	.776**	.886**	.831**	0.27	0.317	21	0.316	0.152	0.373	−0.001	0.122
	V	24	.636**	.667**	.756**	0.092	0.115	24	.567**	.407*	.499*	0.222	0.292
	Zn	24	.551**	.547**	.686**	0.082	0.143	24	.441*	.415*	.476*	0.116	0.139
	TotalPAH	24	0.344	.414	.623**	−0.326	−0.229	24	−0.269	.-435*	.475*	.-675**	.-610**
PC3	C1	24	.863**	.724**	.683**	.697**	.702**	24	.551**	0.306	0.115	.462*	.559**
	F	21	.927**	.888**	.777**	.665**	.656**	18	0.463	0.254	.553*	0.019	0.101
	Cu	24	.497*	.570**	.583**	0.184	0.211	24	.810**	.763**	0.299	.629**	.641**
PC4	Universal bacterial assay	24	.820**	.671**	.674**	.567**	.599**	24	.847**	.589**	−0.188	.831**	.843**
Other	Genotoxic PAH	24	0.25	0.326	.557**	.-423*	−0.332	24	−0.269	−0.392	.477*	.-685**	.-636**
	EU PAH	24	0.131	0.22	.497*	.-533**	.-444*	24	−0.316	−0.353	.489*	.-710**	.-683**
	*Penicillium sp./Aspergillus sp.*	24	.666**	.565**	.571**	.472*	.475*	24	.788**	.659**	−0.045	.778**	.792**
	*Cladosporidium cladosprioides*	24	.787**	.685**	.483*	.820**	.816**	21	.806**	.666**	0.197	.803**	.766**
	*Streptomyces sp.*	24	.550**	.650**	.609**	0.083	0.065	15	.700**	.621*	.668**	0.232	0.361
	*Mycobacterium sp.*	24	.843**	.701**	.682**	.597**	.623**	15	.879**	.900**	0.479	.957**	.982**

The values are Spearman correlation coefficients (ρ), (** *p* < 0.01, * *p* < 0.05).

Genotoxic PAH compounds according to WHO (1998).

EU PAH compounds according to the EU Directive 2014/107/EC.

All of the soil-derived chemical constituents in PC1 correlated positively with cytotoxicity in macrophages in both indoor and outdoor air samples. However, the apoptotic activity correlated positively with most of the indoor air PC1 constituents, whereas in the outdoor air, no such effect was seen. The reason behind this difference remains unclear, but one explanation could be that differences in outdoor and indoor conditions may have influenced the surface characteristics of the mineral particles, because of e.g. stronger condensation occurring outdoors. In addition, most of the soil related chemical constituents showed statistically significant positive correlations with inflammatory activity. Nickel had also a positive correlation with inflammation, although this was not found to be statistically significant.

In PC2 group of constituents, indoor air NO_3_, V and Zn showed clear positive correlations with measured cytotoxic activity, whereas SO_4_ and total number of PAHs had clearly lower associations. In the outdoor air, the detected ions had no effect on the cytotoxic potency of the particles. Instead, levels of V and Zn correlated with increased cytotoxicity and the total number of PAHs also decreased cell viability. In indoor air samples all of the constituents in the PC2 group displayed statistically significant positive correlations with apoptotic activity, but in outdoor air samples only V, Zn and PAHs had a significant association with apoptosis. PAH compounds had a clear negative correlation with the inflammatory markers in the outdoor air samples, whereas in indoor specimens the effect was not so clear. In addition, the SO_4_ concentration also showed a negative association with the inflammatory activity induced by outdoor air particles.

The cytotoxicity induced by the PC3 group of constituents exhibited stronger positive correlations with indoor air particles than with corresponding outdoor air particles. Similarly, the indoor air particles of group PC3 correlated positively with the number of apoptotic cells, whereas their role in the outdoor air was inconsistent. Cl was the only constituent that exhibited any significant positive correlation with the inflammatory markers both indoors and outdoors. The F concentration in the indoor samples had a positive correlation, whereas Cu had no clear effect. In contrast, in outdoor air, Cu revealed a positive correlation with inflammatory activity, whereas F had no clear effect. In summary, this may indicate that the source of the chemicals in the PC3 group is mostly originating from indoors while if they are present outdoors, their role is not so obvious.

Similarly to PC1, all of the microbes both indoors and outdoors showed significant positive correlations with the cytotoxicity markers. Moreover, the increase in the number of apoptotic cells correlated positively with indoor air samples, whereas in the outdoor air samples only *Streptomyces sp*. showed a similar correlation. All the microbes, with the exception of *Streptomyces sp*. displayed a statistically significant positive correlation with the levels of the inflammatory marker both indoors and outdoors. Similarly to the responses induced by PC1, it is possible that differences in the outdoor and indoor air conditions, especially humidity, may explain some of the noted differences, i.e. some of the microbes produce more toxins under dry conditions, and this is a factor which may influence especially the particles toxicological potency of those specimens living indoors.

Overall, there were smaller differences in the indoor air than in outdoor air correlations, which is due to the fact that the strong seasonal variation in the ambient air quality is not so apparent indoors, because of the filtered intake air. In particular, the PAH compounds were more prevalent in the outdoor air, which also exerted a clear immunosuppressive effect as indicated by decreased cytokine production, as well as in disturbed cell cycle and increased number of apoptotic cells. Similar effects have been observed in many of our previous studies with ambient air particles [6-8,33,35]. The role of PAH compounds in this study was smaller in the indoor air. However, in general there can be also significant PAH sources indoors, depending on what activities are being conducted e.g. frying food, smoking and burning wood in a stove with insufficient ventilation conditions. Moreover, exhaust gases released close to the house air intake due to the usage of gardening machines and starting of car in the yard (especially in wintertime) may be important sources for PAHs, as well as small-scale wood combustion in the neighborhood with delayed photochemical cleavage of organic compounds.

#### Comparison between coarse and fine size range

In the search for possible sources of chemical constituents responsible for induced toxicological effects, we also analyzed the coarse and fine size range particles separately. The calculated coefficients for correlations of both size ranges with respect to the measured responses are shown in Table 4.

**Table 4:** **Association between inorganic soluble chemical constituents, polyaromatic hydrocarbons (PAHs) and microbes in PM**_**10–2.5**_ **and PM**_**2.5–0.2**_ **size ranges separately and the inflammatory and cytotoxic response markers in mouse macrophages after exposure to three different doses of particles (50, 150 and 300 μg/ml) for 24 h**

		**PM**_**10–2.5**_						**PM**_**2.5–0.2**_					
		**n**	**MTT**	**PI**	**SubG**_**1**_	**TNFα**	**MIP2**	**n**	**MTT**	**PI**	**SubG**_**1**_	**TNFα**	**MIP2**
PC1	Al	24	.474*	.435*	0.256	.776*	.805**	24	.690**	.577**	0.153	.661**	.635**
	Ca	24	.583**	.503*	.453*	.687**	.763**	24	.810**	.673**	.430*	.623**	.684**
	Fe	21	0.612**	.605**	0.396	.743**	.714**	24	.444*	0.337	−0.043	.542**	.471*
	K	24	.784**	.734**	.564**	.878**	.875**	24	.667**	.572**	0.316	.463*	.489*
	Mg	24	.525**	.503*	0.329	.775**	.810**	24	.863**	.819**	.425*	.717**	.754**
	Na	24	.544**	.498*	0.323	.740**	.790**	24	.823**	.649**	0.352	.613**	.642**
	Ni	24	.619**	.560**	0.396	.806**	.802**	24	.573**	.438*	0.216	0.385	0.388
PC2	SO_4_	24	.695**	.643**	.668**	0.287	0.343	24	.711**	.487*	0.332	.454*	.463*
	NO_3_	24	0.32	0.354	0.281	0.303	0.402	21	.606**	.451*	0.399	0.248	0.264
	V	24	.556**	.536**	.483*	.465*	.500*	24	.607**	.454*	.423*	0.244	0.29
	Zn	24	.688**	.593**	.597**	.648**	.692**	24	.771**	.657**	.503*	.501*	.557**
	TotalPAH	24	0.358	0.298	.519**	0.018	0.13	24	0.379	0.083	0.343	−0.064	−0.011
PC3	Cl	24	.444*	0.35	.553**	0.136	0.242	24	.776**	.575**	.453*	.518**	.586**
	F	18	.889**	.769**	.957**	0.176	0.271	21	.775**	.604**	.548*	0.373	.457*
	Cu	24	.624**	.573**	.423*	.790**	.806**	24	.751**	.814**	.502*	.618**	.654**
PC4	Universal bacterial assay	24	.921**	.890**	.781**	.713**	.680**	24	.634**	0.317	0.159	0.403	.494*
Other	Genotoxic PAH	24	0.393	0.345	.538**	0.062	0.168	24	.406*	0.109	0.365	−0.038	0.017
	EUPah	24	.414*	0.364	.609**	−0.002	0.096	24	0.401	0.108	0.355	−0.02	0.034
	*Penicillium sp./Aspergillus sp*	24	.786**	.816**	.737**	.609**	.611**	24	.503*	0.3	0.089	.429*	.479*
	*Cladosporidium cladosprioid*	24	.603**	.729**	0.337	.857**	.835**	21	.734**	0.416	0.294	.487*	.497*
	*Streptomyces sp.*	21	.765**	.755**	.832**	0.19	0.17	18	.571*	.511*	0.302	0.42	0.437
	*Mycobacterium sp.*	24	.878**	.891**	.733**	.810**	.814**	15	.654**	.529*	0.504	0.474	.565*

The values are Spearman correlation coefficients (ρ), (** *p* < 0.01, * *p* < 0.05).

Genotoxic PAH compounds according to WHO (1998).

EU PAH compounds according to the EU Directive 2004/107/EC.

Soil related constituents of group PC1 were associated with increased cytotoxicity in both the coarse and fine size ranges. However, there was no clear trend with the PC1 group constituents to cause apoptosis in the exposed macrophages. Instead, all of them in coarse size range showed a statistically significant positive correlation with the inflammatory activity. Only nickel displayed no significant correlation in fine size ranges soil related (PC1) constituents. The ability of coarse particles originating from soil to induce cytotoxicity and inflammation has been observed in many previous studies both *in vitro* and *in vivo* [8,10,35,41,44]. A similar effect was also seen with fine particles, even though their role in PM_2.5–0.1_ size range is smaller than that of the larger particles.

The chemical constituents belonging to PC2 group were associated with cytotoxicity in both size ranges, excluding NO_3_ in coarse size range and PAHs in both size ranges. In the coarse size range, all of the constituents, with the exception of NO_3_, correlated with increased apoptotic activity. Instead, in the fine size range, only V and Zn displayed a significant positive correlation with SubG_1_ phase cells. V and Zn increased significantly the level of markers of inflammation in coarse particles, whereas this was found for the SO_4_ and Zn in fine particles. Interestingly, the total sum of PAH-compounds had no significant effect on cell death or inflammatory activity according to the Spearman analysis.

Most of the PC3 group constituents exhibited significant correlations with cytotoxicity and the number of apoptotic cells in both size ranges of PM samples. With respect to the inflammatory response, only Cu showed a significant correlation in both size ranges, whereas Cl correlated with inflammatory activity only in the fine PM samples. However, the largest concentrations of Cl were measured in the coarse PM samples while F was most prevalent in the coarse particles present in the indoor air. Therefore, it is most possible that these correlations are a surrogate effect attributable to some other source.

The microbes showed clearly stronger statistically significant correlations with the measured toxicological parameters in the coarse size-range. This finding was anticipated, because most of the microbial components are known to be present in the coarse size range [28]. Microbes were also found in the fine size range, because microbial fragments and some spores of microbes are smaller than 2.5 μm, and this was used as a cut-off point for the separate size classes.

#### Comparison between seasons

The variation between the chemical constituents and induced toxicological responses were calculated using the whole database. However, due to the limited number of the samples, it was not possible to separate indoor and outdoor samples nor different size ranges from each other in this analysis. The results of this analysis are shown in Table 5.

**Table 5:** **Association between inorganic soluble chemical constituents, polyaromatic hydrocarbons (PAHs) and microbes in different seasons and the inflammatory and cytotoxic response markers in mouse macrophages after exposure to three different doses of particles (50, 150 and 300 μg/ml) for 24 h**

		**Winter**						**Spring**					
		**n**	**MTT**	**PI**	**SubG**_**1**_	**TNFα**	**MIP2**	**n**	**MTT**	**PI**	**SubG**_**1**_	**TNFα**	**MIP2**
PC1	Al	12	0.273	0.07	0.154	0.126	0.245	12	.692*	0.503	−0.049	.951**	.937**
	Ca	12	0.49	0.385	0.231	0.147	0.287	12	.860**	.769**	0.315	.909**	.937**
	Fe	9	0.383	0.017	−0.183	0.633	0.533	12	.713**	0.517	−0.028	.888**	.860**
	K	12	.601*	.720**	.671*	0.035	0	12	.881**	.776**	0.252	.825**	.825**
	Mg	12	0.161	0.091	0.105	−0.175	−0.014	12	.818**	.720**	0.196	.916**	.930**
	Na	12	0.378	0.329	0.336	−0.035	0.084	12	.853**	.748**	0.189	.832**	.818**
	Ni	12	0.053	0.256	0.572	−0.509	−0.484	12	.911**	.795**	0.385	.725**	.736**
PC2	SO_4_	12	0.28	0.538	.818**	−0.315	−0.392	12	0.014	0.105	0.224	−0.524	−0.538
	NO_3_	12	0.035	−0.049	0.203	−0.14	−0.028	12	.650*	0.545	0.147	0.42	0.378
	V	12	.587*	.699*	.727**	0.098	0.063	12	.755**	.678*	0.196	.587*	0.566
	Zn	12	0.252	0.399	0.552	−0.329	−0.294	12	.881**	.797**	0.538	.622*	.664*
	TotalPAH	12	−0.063	0.098	0.483	−0.517	−0.455	12	0.112	0.147	0.315	−0.483	−0.49
PC3	Cl	12	.720**	0.434	0.035	.783**	.853**	12	.820**	.753**	0.305	.893**	.918**
	F	9	.933**	.917**	.717*	.683*	0.6	9	.733*	0.633	.750*	0.183	0.3
	Cu	12	0.2	0.273	0.375	−0.319	−0.228	12	.846**	.811**	0.42	.797**	.825**
PC4	Universal bacterial assay	12	.776**	.692*	0.147	.608*	.594*	12	.909**	.769**	0.315	.874**	.888**
Other	Genotoxic PAH	12	−0.084	0.14	0.566	.-580*	−0.545	12	0.112	0.147	0.315	−0.483	−0.49
	EUPah	12	−0.105	0.154	.615*	.-636*	.-622*	12	0.046	0.091	0.368	.-602*	.-592*
	*Penicillium sp./Aspergillus sp.*	12	.818**	.713**	0.161	.650*	.657*	12	.706*	.664*	.594*	.676*	.655*
	*Cladosporidium cladosprioides*	9	.850**	.917**	.767*	0.3	0.283	12	.708*	0.508	0.077	.676*	.655*
	*Streptomyces sp.*	6	.943**	.943**	0.771	.829*	.829*	12	0.406	0.455	0.538	−0.105	−0.056
	*Mycobacterium sp.*	9	.817**	.900**	.750*	0.233	0.183	12	.783**	.692*	0.259	.944**	.965**
		**Summer**						**Autumn**					
		**n**	**MIT**	**PI**	**SubG1**	**TNFa**	**MIP2**	**n**	**MIT**	**PI**	**SubG1**	**TNFa**	**MIP2**
PC1	A	12	.805**	.851**	0.341	.886**	.939**	12	.636*	.678*	0.301	.958**	.951**
	Ca	12	.755**	.846**	0.49	.748**	.832**	12	.804**	.825**	.741**	.678*	.720**
	Fe	12	.692*	.755**	0.287	.832**	.888**	12	0.462	.594*	0.175	.888**	.881**
	K	12	.587*	.731**	0.476	0.517	.629*	12	0.573	.671*	0.552	.622*	.650*
	Mg	12	.881**	.937**	.615*	.720**	.797**	12	.853**	.867**	.601*	.916**	.944**
	Na	12	.881**	.916**	.741**	.580*	.657*	12	.734**	.797**	.594*	.804**	.832**
	Ni	12	.587*	.720**	0.538	0.448	0.573	12	0.442	0.495	0.495	0.431	0.452
PC2	SO_4_	12	−0.168	−0.007	0.469	−0.503	−0.378	12	0.119	0.105	0.559	−0.469	−0.413
	NO_3_	12	0.462	0.559	.650*	0.119	0.203	9	0.45	.733*	0.383	.667*	.750*
	V	12	0.399	0.559	0.448	0.301	0.448	12	0.52	.671*	.608*	0.485	0.527
	Zn	12	0.231	0.413	0.517	0.014	0.168	12	0.545	.587*	.790**	0.126	0.196
	TotalPAH	12	0.186	0.34	.669*	−0.224	−0.095	12	−0.021	0.112	0.455	−0.441	−0.378
PC3	C1	12	.811**	.846**	.615*	.601*	.657*	12	.762**	.825**	0.573	.867**	.895**
	F	12	.797**	.832**	.881**	0.308	0.371	9	.750*	.733*	0.633	.733*	.783*
	Cu	12	0.462	.594*	.769**	0.091	0.238	12	.818**	.769**	.741**	.643*	.678*
PC4	Universal bacterial assay	12	.916**	.846**	0.28	.944**	.923**	12	.958**	.818**	.783**	.650*	.692*
Other	GenoPAH	12	0.042	0.203	0.545	−0.308	−0.175	12	−0.056	0.056	0.427	−0.503	−0.441
	EUPah	12	−0.278	−0.091	0.344	−0.499	−0.359	12	−0.112	−0.021	0.378	−0.573	−0.51
	*Penicillium sp./Aspergillus sp.*	12	.916**	.846**	0.28	.944**	.923**	12	0.545	.713**	0.517	.643*	.678*
	*Cladosporidium cladosprioides*	12	.916**	.846**	0.28	.944**	.923**	12	0.573	.713**	0.301	.909**	.923**
	*Streptomyces sp.*	12	.797**	.846**	.748**	0.469	0.552	9	.867**	.983**	.800**	0.533	0.65
	*Mycobacterium sp.*	9	.900**	.883**	0.25	.967**	.967	9	.733*	.917**	0.567	.817**	.883**

The values are Spearman correlation coefficients (ρ), (** *p* < 0.01, * *p* < 0.05).

Genotoxic PAH compounds according to WHO (1998).

EU PAH compounds according to the EU Directive 2004/107/EC.

PC1 group constituents had a positive statistically significant correlation especially with cytotoxicity and inflammation during spring, summer and autumn. Instead, during the wintertime this phenomenon was not seen. This was mostly because of snow coverage limiting resuspension of dust from the soil as well as road-dust particles at the wintertime. However, the soil related constituents also increased the inflammatory activity in the indoor air; this may have been due to airing of the house by opening windows and doors during warm seasons. This is a relatively common way to cool down the indoor air temperature during summertime in the buildings located in south borealic climatic zone, where houses are not usually equipped with air conditioners. Moreover, it is possible that small amounts of sand are transported indoors in shoes and thereafter become resuspended in the indoor air. One possible source for soil related constituents in indoor air is also cat litter, because there were three cats living in the test household [28].

The chemical constituents in group PC2 showed both positive and negative correlations with the measured toxicological responses. V and Zn exhibited their strongest correlation with cytotoxicity in the springtime, whereas the total sum of PAH compounds displayed no clear seasonal-related variation in its cytotoxicity. In both winter and summer these compounds were seen to increase number of apoptotic cells. On the other hand, they had a negative association with the inflammatory activity, but this trend achieved statistical significance only in the winter and spring samples.

Many of the correlations between toxicological end points and the PC3 group constituents were statistically significantly positive. Only Cu in the wintertime revealed inconsistent correlations with the measured parameters. However, the PC3 group constituents were not linked to any known source in this experiment, and therefore their role remains uncertain.

There were no clear seasonal differences between the ability of microbes to induce cell death, apoptosis or inflammation in mouse macrophages. One explanation for this is that some of the microbes occurred more likely in the indoor air samples during cold seasons, but they had their highest concentrations outdoors during warm seasons. Kaarakainen et al. [45] showed that microbe numbers in PM_10_ were the highest during autumn and summer. Bacteria had a clear role in inducing cytotoxicity and inflammation during all seasons, whereas their role in apoptosis was significant only in the autumn. The responses linked to bacteria tended to follow the same trend as found with soil related constituents in the PC1 group. It is highly possible that bacteria are transported along with these crustal-derived particles, especially in the coarse size range. Other microbes displayed inconsistencies in their correlations with toxicological end points in the different seasons. It should be taken into account that in this analysis both outdoor and indoor air samples were analyzed in the same group in order to obtain large enough number of measurements. Therefore, there was most probably some mixing factor when seasonal variation was being evaluated.

## Conclusions

The present study revealed that the most important sources for induced toxicological health related effects were associated with soil-derived constituents, local combustion emissions and microbes. The outdoor sources and road dust episode had a significant role in determining indoor air quality, particularly during the warm seasons, when the significance of outdoor microbes was also greater in the indoor air. In contrast, during wintertime, the role of indoor sources of the particles and microbes was more significant. In the wintertime, the local combustion emissions also played a more significant role in both indoor and outdoor air, in comparison to the other seasons.

Even though there were clear seasonal differences in the abilities of indoor and outdoor air to induce inflammatory and cytotoxic responses, there were relatively small differences in the chemical composition of the particles responsible for those effects. It is possible that this is a special feature of a relatively good air quality environment and the fact that the studied house had been newly built. Its modern mechanical exhaust and air supply as well as well insulated and tight insulation materials together may also possibly explain the difference between the roles of indoor and outdoor air sources. During the wintertime the doors and windows are kept closed whereas during warm seasons in buildings without air conditioning, ventilation is usually enhanced by opening windows to allow outdoor air to come into the building.

## Material and methods

### Particulate sampling and PM sample preparation

The experiments were conducted in a suburban area of a Finnish city that is home to approximately 95,000 inhabitants. The indoor air samples were collected from a new single family house that has wooden frame and a wooden covering with a total living-area of 140 m^2^ [28]. The house represents modern building technology, where energy saving materials have been used especially for heat insulation. The house was equipped with a modern mechanical exhaust and air supply (Ilto 440) with a heat recovery system. All of the intake air was passed through a EU7 class air filter, with the exception of the air entered into the house when doors and windows were opened, at especially on warm days. The air exchange rate in the house was set to 0.64 air changes per hour. The house was connected to district heating, but had also a log- fired masonry heater. The size-segregated particulate samples were collected continuously with the modified high volume cascade impactor (HVCI) [46] adjusted to 850 l min ^−1^ from the main exhaust line before the heat exchanger. Thereafter, the air was circulated back to the house ventilation system. The sampling instruments were located in the attic, which was not connected to the house ventilation system. The house was home to 2 adults, 1 child and 3 cats; the adults kept a diary of their activities to reveal possible aerosol sources. The possible sources for indoor particles included frying food, sauna heating (an electric stove), cleaning and heating of the masonry heater. Simultaneously, outdoor air samples were collected throughout the sampling campaigns with a separate identical impactor at a transportable sampling station, which was located in the backyard area with no streets in its vicinity. Local sources for outdoor air particles included houses with domestic-scale biomass-fired combustion appliances, nearby streets with very low traffic volumes, starting of the car on the yard, earthworks in the neighboring lots and other point sources.

Four separate sampling campaigns were performed during a one year period i.e. one in each season (Winter 25.3.-19.3.2010; Spring 20.5.-3.6.2010; Summer 19.7.-8.8.2010; Autumn 30.9.-21.10.2010). The PM samples representing coarse (PM_10–2.5_) and fine (PM_2.5–0.2_) size ranges were collected on polyurethane foam substrates. The prevailing weather conditions and collected size-segregated particle masses during the experiment have been reported by Sippula et al. [28]. Prior to use, all substrates were washed with methanol to remove all possible impurities. Then all the substrates were weighed before and after sample collection with an analytical balance (Mettler Toledo XP 105DR, Mettler Instrumente AG, Zurich, Switzerland) and the collected particulate mass was calculated. The effects of the surrounding temperature, pressure and humidity were corrected by using control substrates and appropriate conditioning times (24 h). Thereafter, the particles were extracted from the substrates by using a methanol extraction method [38]. Briefly, the substrates were placed into a 50 ml glass tube that was filled with methanol (J.T. Baker HPLC grade, Deventer, The Netherlands) and treated in a water bath sonicator (FinnSonic m20, FinnSonic Ltd., Lahti, Finland) for 2× 30 min at a temperature not exceeding +35°C. Thereafter, the samples were pooled together according to size range and excess methanol was evaporated at +35°C in a rotary evaporator (Heidolph laborota 4000) set at 150 mbar, and chiller (Lauda WK500). Finally, the concentrated suspension was divided into 10 ml glass tubes as defined amounts of particulate mass and dried under nitrogen (99.5%) flow at +35°C on a heat block. The dried particulate samples were stored at −20°C. The extraction efficiency of the collected particle mass was 91%. The blank filter samples were prepared in a corresponding volume. Subsequently, the suspension was diluted in pyrogen-free water to obtain the final concentrations that were used in the cell exposures.

### Characterization of the samples

The chemical analyses of the particle samples have been described in detail by Sippula et al. [28]. Shortly, the elemental composition of the particulate samples (total 32 elements) was analyzed in an inductively coupled plasma-mass spectrometer (ICP-MS) and water soluble anions (total 6 anions) by ion chromatography (IC). A total of 30 polycyclic aromatic hydrocarbons (PAH) compounds were measured in a gas-chromatograph mass spectrometer (6890 N GC, equipped with an inert Mass Selective Detector, Agilent Technologies) and a HP-17-MS column for the separation of the compounds.

Before the qPCR analyses of microbes, DNA was isolated from the samples by using NucleoMag 96 Plant (Macherey-Nagel, Duren, Germany) DNA isolation kit and the KingFisher mL automated DNA isolation station (Thermo Fisher Scientific) according to the kit manufacturer’s instructions. Purified DNA samples were stored at −80°C until analyses. In the microbial analyses, concentrations of two fungal and two bacterial groups and total bacteria were determined using qPCR method. The assays used to detect the fungal genera Penicillium/Aspergillus/Paecilomyces variotii (PenAsp), the fungal species Cladosporium cladosporioides (Clad1), the bacterial genera Mycobacterium spp. (Myco) and Streptomyces spp. (Strep) and the universal bacterial assay have been previously validated and published [47-50]. The qPCR cycling was done using either the Rotor-Gene RG-3000 (Corbett Life Science, Australia) or ABI Prism 7000 (Applied Biosystems) instrument following the cycling conditions given in the original publications.

### Cell exposure

The mouse RAW264.7 macrophage cell line (ATTC, Rockville, MD, USA) was used in the exposures. The cells were cultured at +37°C in 5% CO_2_ atmosphere in RPMI 1640 –medium (Gibco, Paisley, UK) supplemented with 10% of heat inactivated fetal bovine serum (FBS) (Sigma, St. Louis, MO, USA), 2 mM L-glutamine and 100 U/ml penicillin-streptomycin (Gibco, Paisley, UK). The cell suspension (5× 10^5^ cells/ml) was dispensed into 6-well plates (Costar, Corning Inc., NY, USA). The cells adhered for 24 h and then the fresh complete medium was changed 1 h before the exposure. The macrophages were exposed to particulate samples for 24 h to three doses (50, 150 and 300 μg/ml) or to the corresponding blank sample at a 150 μg/ml dose. Three independent exposures were made in duplicate.

After the exposure, the macrophages were gently scraped from the well bottoms with a cell lifter and resuspended into cell culture medium. Cell viability was assessed immediately after the end of the exposure using the MTT-test. The remaining cell suspension was centrifuged (6.082 g for 5 min at +4°C, Heraeus Biofuge Fresco) to separate the cells and the medium, which was then stored at −80°C for the subsequent cytokine analyses. The cell pellets were suspended into phosphate buffered saline (PBS) and half of them were prefixed with ethanol (70% v/v) and stored at +4°C for detection of apoptosis and cell cycle stage by DNA content analysis. The other half portion of the cells was kept fresh for use in the propidium iodide (PI)-staining.

### Analysis of toxicological responses

The concentrations of proinflammatory cytokine TNF-α and chemokine MIP-2 were analyzed from the cell culture medium using enzyme-linked immunosorbent assay (ELISA) kits (R&D Systems, MN, USA) following slightly modified manufacturer’s instructions (Jalava et al. [38]). Absorbance was measured using a microplate reader (Victor^3^, PerkinElmer, Finland) at wavelength 450 nm and analyzed by WorkOut software version 2.0 (Dazdaq Ltd., Brighton, UK).

The mitochondrial activity of the macrophages was estimated using the MTT-test and the viability of the cells was calculated as a percentage by comparing absorbances from cell suspensions exposed to particulate samples with those from the corresponding control cells (Happo et al. [29]). Optical density was measured with a microplate reader (Victor3, PerkinElmer, Finland) and analyzed by WorkOut software version 2.0 (Dazdaq Ltd., Brighton, UK).

Cytotoxicity was also estimated by flow cytometry detecting the PI-positive cells with a lowered cell membrane potential. Briefly, the cell suspension was centrifuged (5 min at 370 g) to separate the cells from the culture medium and washed once with 1 ml of phosphate-buffered saline (PBS). Thereafter, the cells were resuspended into 0.5 ml of PBS and 1 μg/ml of PI was added. The samples were incubated for 15 min in dark at room temperature before flow cytometric analysis (CyAn ADP, Beckman Coulter, USA). A total of 10,000 cells per sample were analyzed with Summit software version 4.3.

Cellular DNA content and thus the cell cycle stage of non-apoptotic cells were analyzed by PI staining of permeabilized cells as earlier described [29]. This method provides information about the cell cycle, and apoptotic cells can be identified as cells containing fragmented DNA (number of hypodiploid cells as in sub G1 peak) [51-53]. A total of 10,000 cell counts per sample were collected with a Cyan ADP flow cytometer using an emission wavelength of 613 ± 20 nm and thereafter analyzed by using Summit software version 4.3. The gate was set to discriminate at least 80% of the particulate matter outside of the analysis.

### Statistical analyses

All of the data were analyzed using the IBM Statistics 21.0 (IBM ®, New York, NY, USA). First, all the measured toxicological responses were analyzed with Levene’s test for equality of variances before analyzing the data with the analysis of variance (ANOVA) and Dunnett’s test. In cases where Levene’s test gave values < .05, then the Kruskal-Wallis test was applied. The differences in data were regarded as statistically significant at *p* < .05. Differences between seasons were tested by Tukey’s HSD or Dunnett’s C test. Statistically significant differences between the corresponding indoor and outdoor air samples were analyzed with two-tailed Mann–Whitney test (*p* < .05). Measured values in the groups that gave statistically significant differences (PM_10–2.5_ and PM_2.5–0.2_ size ranges) were analyzed with Spearman’s rank correlation (two-tailed) to detect the linear relationships between the variables.

When analyzing multivariate dataset with large number of variables, it is often desirable to reduce their dimensionality. Principal component analysis (PCA) is one technique for achieving this goal. It replaces the p original variables with a smaller number, q principal components, which are linear combinations of the original variables. We used PCA to find the components which would encapsulate the information from the large number of inter-correlated quantitative dependent measurement variables. The principal components (PC) are calculated in such a way that the first principal component accounts for as much of the variability in the data as possible, and each succeeding component in turn accounts for as much of the variability as possible under the constraint that it is orthogonal to the preceding components. In the determination of the number of PCs, the cut-off point was an eigenvalue of 1.00 or above. The founded principal components were used as independent variables in linear regression analysis used to detect the relationships between the toxicological responses and the air samples. All those variables for which there were reasonable numbers of data points were tested in the PCA and then the variables with non-significant loadings were removed from the final model.

## Additional files

Additional file 1: Table S1. Concentrations of chemical and microbial constituents in outdoor and indoor particle size-fractions. Chemical species are given in μg/mg and microbes in units/mg. Additional file 2: Table S2. Measured toxicological responses of indoor and outdoor air particles of different size-fractions. MTT refers to the percentage of dead cells when compared to control level. PI exhibits percentage of PI positive cells assessed in PI exclusion test. SubG_1_ and G_2_M show percentage of cells in distinct phases of cell cycle. TNFα and MIP-2 values are measured cytokine concentrations in the medium (ng/ml).
