# Supplementary figures and images for: Role of microbial and chemical composition in toxicological properties of indoor and outdoor air particulate matter
Source: Part Fibre Toxicol. 2014 Nov 25;11:60. doi: 10.1186/s12989-014-0060-6 (PMC4264261; doi:10.1186/s12989-014-0060-6)

## Slide 1
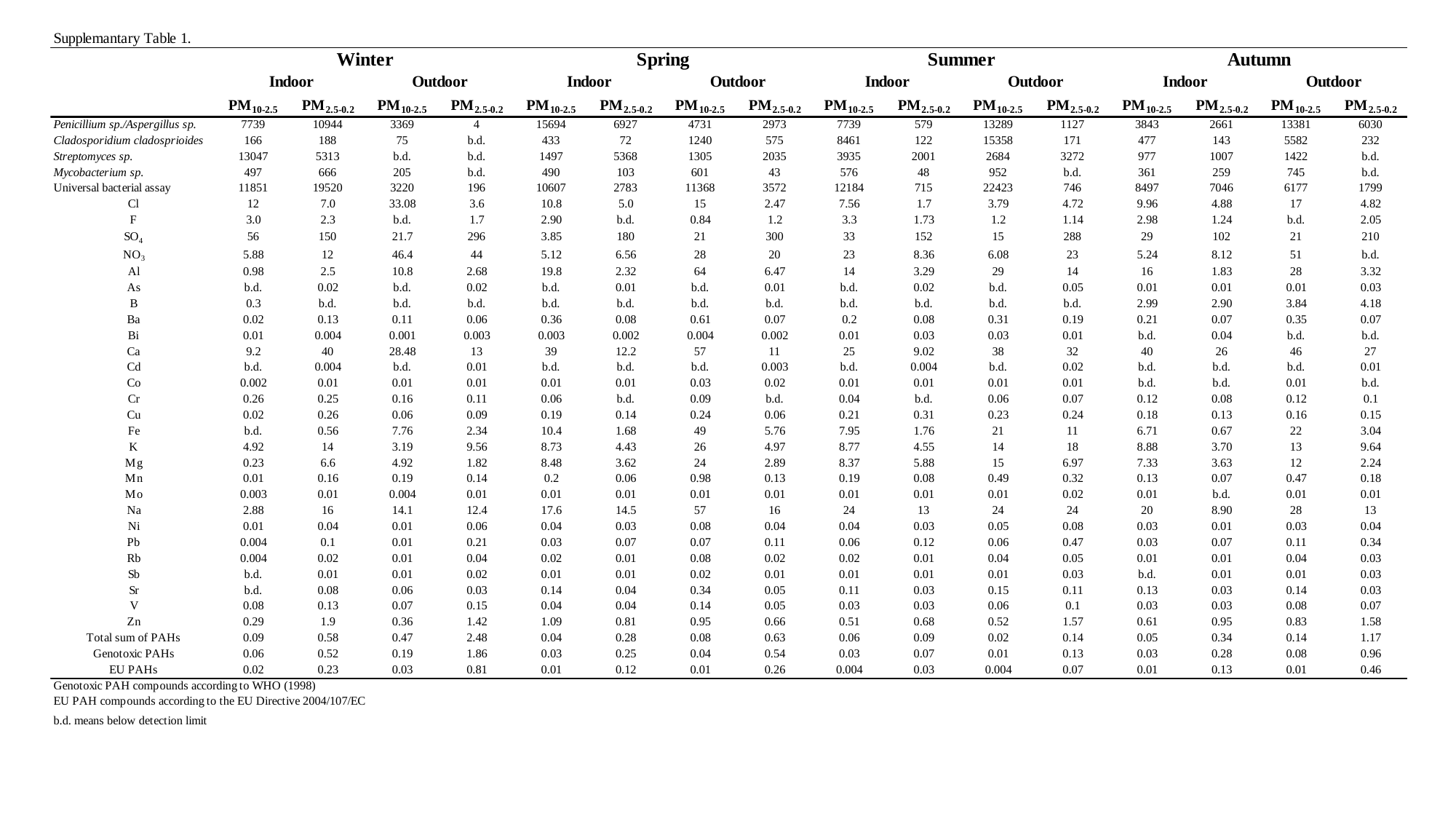

Supplement: Additional file 1: Table S1. — Concentrations of chemical and microbial constituents in outdoor and indoor particle size-fractions. Chemical species are given in μg/mg and microbes in units/mg. [file 12989_2014_60_MOESM1_ESM.pptx]

## Slide 1
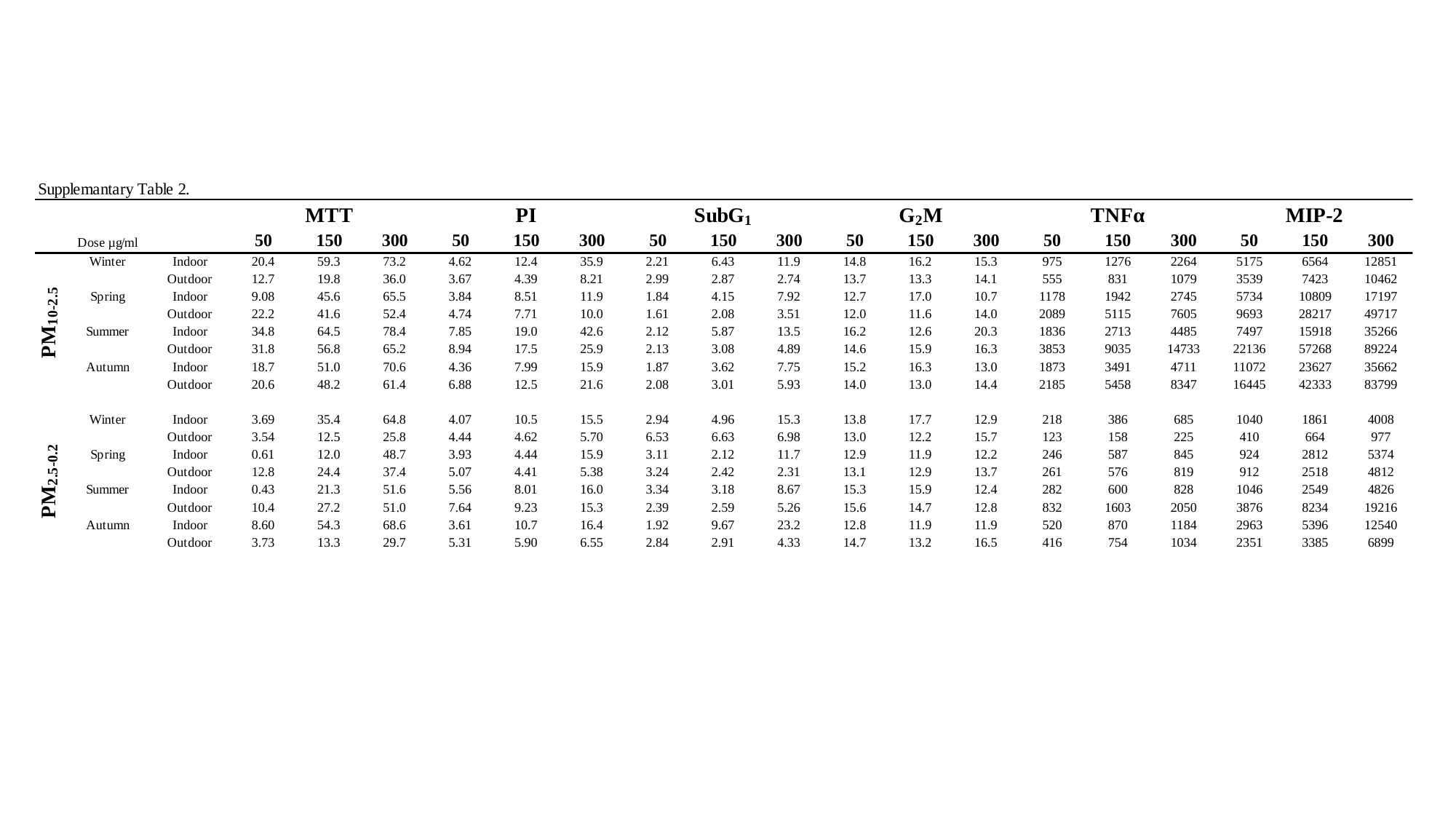

Supplement: Additional file 2: Table S2. — Measured toxicological responses of indoor and outdoor air particles of different size-fractions. MTT refers to the percentage of dead cells when compared to control level. PI exhibits percentage of PI positive cells assessed in PI exclusion test. SubG1 and G2M show percentage of cells in distinct phases of cell cycle. TNFα and MIP-2 values are measured cytokine concentrations in the medium (ng/ml). [file 12989_2014_60_MOESM2_ESM.pptx]
